# Adenovirus Structure: What Is New?

**DOI:** 10.3390/ijms22105240

**Published:** 2021-05-15

**Authors:** José Gallardo, Marta Pérez-Illana, Natalia Martín-González, Carmen San Martín

**Affiliations:** 1 Department of Macromolecular Structures, Centro Nacional de Biotecnología (CNB-CSIC), 28049 Madrid, Spain; jgallardo@cnb.csic.es (J.G.); marta.perez@cnb.csic.es (M.P.-I.); 2 Department of Condensed Matter Physics, Universidad Autónoma de Madrid (UAM), 28049 Madrid, Spain; natalia.marting@uam.es

**Keywords:** adenovirus, structure, cryo-EM, maturation, minor coat proteins, core proteins, crystallography, cryo-electron microscopy

## Abstract

Adenoviruses are large (~950 Å) and complex non-enveloped, dsDNA icosahedral viruses. They have a pseudo-T = 25 triangulation number with at least 12 different proteins composing the virion. These include the major and minor capsid proteins, core proteins, maturation protease, terminal protein, and packaging machinery. Although adenoviruses have been studied for more than 60 years, deciphering their architecture has presented a challenge for structural biology techniques. An outstanding event was the first near-atomic resolution structure of human adenovirus type 5 (HAdV-C5), solved by cryo-electron microscopy (cryo-EM) in 2010. Discovery of new adenovirus types, together with methodological advances in structural biology techniques, in particular cryo-EM, has lately produced a considerable amount of new, high-resolution data on the organization of adenoviruses belonging to different species. In spite of these advances, the organization of the non-icosahedral core is still a great unknown. Nevertheless, alternative techniques such as atomic force microscopy (AFM) are providing interesting glimpses on the role of the core proteins in genome condensation and virion stability. Here we summarize the current knowledge on adenovirus structure, with an emphasis on high-resolution structures obtained since 2010.

## 1. Introduction

Adenoviruses were discovered close to 70 years ago [1], and since then they have been found in all types of vertebrates [2]. As basic science tools, they have provided fundamental insights in biological functions such as splicing [3]. Currently, they are best known by their potential as therapeutic vectors, culminating with their use as SARS-CoV-2 vaccine vehicles during the COVID-19 pandemic [4,5,6]. Adenoviruses soon became an object of interest for structural biologists—they were among the first viruses to be imaged by electron microscopy (EM); their major coat protein was one of the first animal virus proteins to be crystallized; and they were used to demonstrate the possibility of imaging frozen-hydrated biological material in the electron microscope, in the early days of cryo-EM. However, although the general capsid organization was soon unveiled, reaching the finest details of adenovirus architecture required many years of studies, and could only be attained when cryo-EM realized its potential to provide near-atomic resolution structural data [7]. The human adenovirus type 5 (HAdV-C5) virion was by then the largest biological object ever solved at high resolution by any structural biology technique. In fact, a few more years of analyses were required to reconcile the virion models based on cryo-EM and X-ray crystallography data [8]. These historical aspects have previously been reviewed [9]. Here, we summarize the knowledge available after publication of the first high-resolution map [7], and discuss new adenovirus-related structures reported since 2010. These include fiber heads and complete virions from previously uncharacterized species and genera, providing new insights into the structural diversity and receptor binding modes within the *Adenoviridae* family (Table 1).

## 2. Components and Organization of the Adenovirus Virion

**Figure 1:**
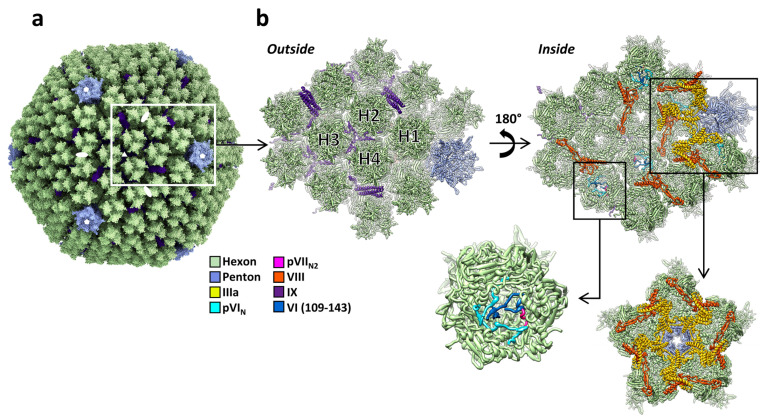
Structure of the AdV capsid. (**a**) General view of the HAdV-C5 capsid [28]. White symbols indicate the position of the icosahedral symmetry axes. The white rectangle highlights one icosahedral asymmetric unit. The fibers are not represented here, as they cannot be traced in studies using icosahedral symmetry. Structure rendering with ChimeraX [36]. (**b**) Zoom in on the asymmetric unit and its closest neighbors. Two views are provided: as seen from outside the capsid (left) and from inside (right). The four unique hexon trimers are labeled H1 to H4, and the different proteins are colored according to the color key at the left. One of the hexons is further magnified to show the pVI_N_ and pVII_N2_ peptides. Additionally highlighted is the GOS (penton plus peripentonal hexons), depicted as seen from inside the capsid. Structures rendered with Chimera [37].

The International Committee for the Taxonomy of Viruses (ICTV) currently recognizes 86 different adenovirus species, grouped in six genera (https://talk.ictvonline.org/ictv-reports/ictv_9th_report/dsdna-viruses-2011/w/dsdna_viruses/93/adenoviridae, accessed on 13 May 2021). Their linear dsDNA genome varies in length between 26 kbp in the frog adenovirus (FrAdV-1, a Siadenovirus) and 48 kbp in the only fish adenovirus isolated so far (WSAdV-1, an ichtadenovirus) [33,34]. This genome is packed inside a *pseudo* T = 25 icosahedral capsid with a diameter of approximately 95 nm vertex to vertex (Figure 1a). The major coat protein, hexon, forms the icosahedron facets. Capsomers at the vertices are formed by penton base and fibers. These two proteins are key players in the initial stages of infection, as they are in charge of cell receptor interaction. A series of minor coat proteins help to assemble and maintain the shell, and have been termed “glue” or “cementing” proteins. In the Mastadenovirus genus, which includes the human adenoviruses, minor coat proteins IIIa, VI, and VIII are located on the inner capsid surface, and protein IX on the outside (Figure 1a,b). The set of minor coat proteins and their organization changes between genera and species. Three of them (IIIa, VI, and VIII) are conserved in all the *Adenoviridae* family and therefore would be expected to play crucial roles during assembly [35]. For example, protein VI is a key factor for AdV entry in the host cell (see Section 3.5 and Section 4.4). The capsid geometry can be represented as a set of two different kinds of tiles. A flat tile corresponding to most of the facet is formed by the nine central hexon capsomers, and is often referred to as “group-of-nine” (GON). The other kind of tile is formed by the penton and its five surrounding hexon capsomers (peripentonal hexons). This tile has been termed “group-of-six” (GOS) [7,9]. The icosahedral asymmetric unit is composed by four hexon trimers, one penton base monomer, one protein IIIa, two copies of VIII, and four copies of protein IX (Figure 1b).

Inside the capsid, a large amount of virus encoded, DNA-binding proteins accompany the adenovirus genome, forming a non-icosahedral core. Some of them (the core proteins) are considered DNA-condensing agents due to their positive charge [38,39]. Others are involved in genome replication (terminal protein), genome packaging (IVa2), or maturation (adenovirus protease, AVP) [40,41,42].

## 3. Structure of the Capsid Proteins

### 3.1. Hexon

**Figure 2:**
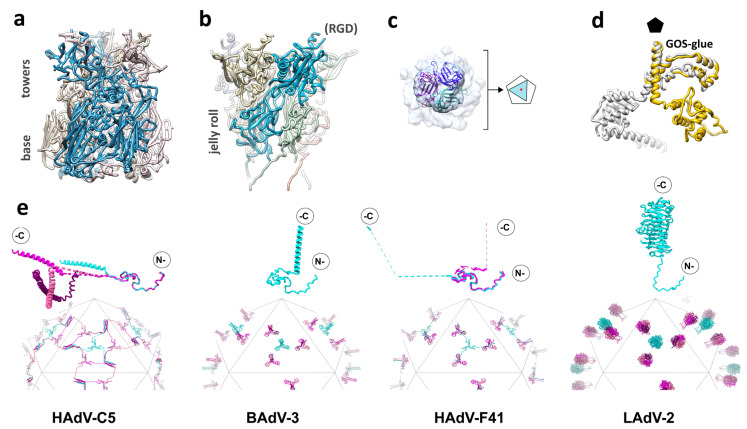
Structure of the AdV capsid proteins. (**a**) Hexon trimer, with one of the monomers highlighted in vivid blue. (**b**) Penton base pentamer. The location of the untraced RGD loop in one of the monomers is indicated. In (**a**,**b**), the inner side of the particle would be at the bottom. (**c**) Localized reconstruction, without symmetry enforcement, of the HAdV-D26 fiber bound to the penton base. The view is from outside the capsid. An atomic model of the knob [19] is fitted into the density. Notice that the knob density appears clearly trimeric. The schematic diagram illustrates how the three-fold symmetric fiber (triangle) is shifted relative to the center (red dot) of the five-fold symmetric penton base (pentagon). Adapted from [47]. (**d**) Comparison between the LAdV-2 (yellow) and HAdV-C5 (gray) protein IIIa structures. Notice that the GOS-glue domains and part of the connecting helix overlap, but the VIII-binding domain in the LAdV-2 protein swings away from its position in the human virus. The black pentagon indicates the position of the 5-fold symmetry axis. Modified from [31]. (**e**) Schematics showing the organization of protein IX in HAdV-C5, BAdV-3, and HAdV-F41, and LH3 in LAdV-2. Molecules forming triskelions located at the center of the facet (I3 symmetry axis) are in cyan, and those located at the L3 axes in several shades of pink. The rope domains in HAdV-C5, and the rope and C-terminal domains in HAdV-F41, are depicted as dashed lines, indicating non-modeled residues. Monomers of protein IX/LH3 in the asymmetric unit are depicted on top of each schematic, with the N- and C-termini indicated. For HAdV-C5 and HAdV-F41, the four monomers in the AU are overlapped according to their triskelion region, to highlight the different conformations of the rope domain. Adapted from [30].

Hexon is the main building block of the adenovirus protein shell, accounting for approximately 60% of the total virion mass. In the capsid there are 720 hexon monomers, organized in 240 trimers, with 12 trimers per facet (Figure 1a,b). Hexon is a large polypeptide, more than 900 amino acids long in all known adenovirus types. The monomer folds as two eight-stranded β barrels, or jellyroll domains, held apart by a small β-sheet [43,44]. The double jelly rolls form the pseudo-hexagonal base of the trimeric hexon capsomer. Long loops intercalated between the β-strands form the hexon towers, and contain the hyper variable regions (HVRs) (Figure 2a) [45]. The N-termini and C-termini adopt different conformations depending on their location in the capsid, to establish interactions between hexons and minor coat proteins IIIa and VIII [7]. The adenovirus capsid is described as pseudo-T = 25 because of the oligomeric arrangement of the hexon. Since hexons are trimers and not hexamers, the icosahedral asymmetric unit is composed of 4 × 3 (hexon molecules) + 1 (penton molecule) = 13 independent polypeptides, instead of the 25 predicted by the Caspar and Klug quasi-equivalence theory [9,46].

The hexon architecture is highly conserved throughout the *Adenoviridae* family. The main differences reside in the HVRs, which present a varying degree of flexibility in the different virus types. In the HAdV-C5 cryo-EM model, only four of the seven loops at the top of each hexon monomer could be traced [28]. In contrast, a recent study of the enteric HAdV-F41 solved all the loops except HVR4 [30]. In human adenoviruses of species C, HVR1 presents a unique, 32 residue-long acidic loop that confers a large negative charge to the outer capsid surface [48]. In HAdV-F41 and HAdV-D26, HVR1 is shorter than in HAdV-C5 and could be fully traced in cryo-EM maps [29,30]. The acidic HVR-1 in HAdV-C5 seems to be involved in electrostatic interactions with neutralizing defensins, and its absence in species D and F may play a role in determining the enteric or ocular tropism of these viruses, although this aspect is not well understood yet [30,49]. In lizard adenovirus type 2 (LAdV-2), an Atadenovirus, the loops are shorter and could be modeled without gaps. It has been proposed that simpler loops could correlate with lower evolutionary pressure induced by the immune system in reptiles [31]. In HAdV-C5, the valley formed by the three hexon towers is involved in interactions with coagulation factors [50]. Most recently, this region has also been shown to bind the cell receptor CD46, revealing a new entry mechanism for a large group of viruses in HAdV species D [12].

### 3.2. Penton Base

Pentamers of penton base fill the gap left by the five peripentonal hexons. The penton base protein folds into two domains: a single jellyroll and an upper insertion facing the solvent-exposed exterior (Figure 2b) [44,51]. This upper domain contains the hypervariable loop, which due to its flexibility, has not been traced in any of the available HAdV structures [7,27,29,30,51]. In most human adenoviruses, the hypervariable loop bears the RGD sequence, an α_v_ integrin-binding motif. Enteric HAdV-F40 and HAdV-F41 lack the RGD motif, having instead RGAD and IGDD [52]. Nevertheless, it has been recently shown that HAdV-F41 binds laminin-binding integrins [53]. Since the RGD loop is also involved in neutralization by the enteric defensin HD5, it has been proposed that lack of both this sequence motif and the acidic HVR1 in hexon may play a role in facilitating infection of intestinal cells by HAdV-F40 and HAdV-F41 [30,49]. Another surface-exposed variable loop presents sequence divergence and different conformations in the human adenoviruses [7,29,30,51]. This loop, whose role is unknown, has been proposed as a site to be engineered for gene therapy, along with the hypervariable loop [51,54]. In human adenoviruses, a long (~50 residues) N-terminal arm extends away from the main body of the protein towards the interior of the virion. Only those arm residues closest to the jelly roll domain (residues 37-51 in HAdV-C5) are ordered, and interact with two monomers of the inner coat protein IIIa [7,29]. The rest are disordered, and seemingly plunge into the non-icosahedrally ordered core [7]. This disordered part is absent in Atadenoviruses, which have a shorter penton base protein and also lack the mobile, variable loops on the outer surface, as observed for the hexon HVRs [31]. Comparison of a high-resolution structure of recombinant HAdV-F41 penton base with the same protein in the context of the virion has shown that regions of the protein involved in interactions with the peripentonal hexons, fiber, and protein IIIa are disordered in solution, but become ordered upon capsid assembly [27]. Virus-like particles can be formed by penton base pentamers of certain HAdV species assembling in dodecahedra, with uses in receptor identification, gene delivery, and vaccine development [55].

### 3.3. Fibers

Trimeric fibers are attached to the outer surface of the penton base pentamer, forming a non-covalent complex. Each fiber is composed of three domains: head, shaft, and N-terminal tail. The C-terminal globular head (also named knob) folds as an anti-parallel β-sandwich and is responsible for fiber trimerization and attachment to the receptor at the host cell membrane. Fibers can bind receptors in a non-stoichiometric way, as exemplified by a recent cryo-EM study on the HAdV-B3 fiber head bound to desmoglein-2 [20], where the trimeric head was found to bind either one or two copies of the receptor, but not three. The structural aspects of receptor binding by human adenovirus fiber heads have recently been reviewed [56]. The last years have also provided extensive information on the structures of non-human adenovirus fibers heads, including those for genera not previously analyzed, such as Siadenoviruses and Atadenoviruses (Table 1). Although the general β-sandwich architecture is conserved, these structures show variability in the loops connecting the β-strands. The loops are very short in Atadenoviruses, producing the smallest fiber head known so far [17]. The Siadenovirus fiber heads are more similar to those found in reovirus than to other adenoviruses, and the monomer has a unique β-hairpin that embraces the neighboring subunit [14,44].

The central domain of the fiber protein folds as a trimeric β-spiral and forms a shaft of variable length, depending on the virus type [57,58]. Shaft length and flexibility also play a role in AdV entry in the cell, by facilitating virion interaction with both the primary receptor via fiber and integrins via penton base [59]. Recombinant fibers of a minimum shaft length can assemble into stable trimers in the absence of the head [60]. Finally, the extended N-terminal tails, with a FNPVYPY sequence conserved in all human adenoviruses, bind at the cleft formed by two adjacent penton base monomers to attach the fiber to the rest of the capsid. Details on the architecture of the proximal part of the fiber and its attachment to the capsid have been obscured by their lack of compliance with the icosahedral symmetry that cryo-EM studies usually exploit to reach high resolution. The 330 Å long HAdV-C5 fiber is flexible, bending near the surface of the penton base and therefore being blurred out when projections of thousands of particles are averaged. In the cryo-EM structure of HAdV-C5, only density for the lower part of the fiber shaft was observed, suggesting its interaction with a hydrophobic ring at the center of the penton base pentamer [61]. The shorter fibers in species B (130 Å) and D (150 Å) are more rigid, facilitating the visualization of the complete fiber in cryo-EM maps of HAdV-D26 and HAdV-C5 pseudotyped with the HAdV-B35 fiber [29,62]. However, due to the symmetry mismatch between the trimeric fiber and the imposed icosahedral symmetry, in all cases the fiber density displayed an artefactual 5-fold symmetry, with apparently five N-terminal fiber tails bound to the penton surface, when there should be only 3 binding sites occupied [29,61,62]. Fortunately, recent advances in cryo-EM image processing have started providing the means to extricate non-icosahedral details from icosahedral virus capsids [63]. Although at moderate resolution (~7 Å), application of these methods to an adenovirus has shown the disposition of the three N-terminal fiber tails bound to the HAdV-D26 penton base, and the clearly trimeric fiber head (Figure 2c). It has also revealed that the HAdV-D26 fiber shaft is slightly tilted with respect to the penton base [47]. Cryo-EM studies also show how the fiber N-terminal peptides extend further than previously observed by X-ray crystallography [51], wrapping around the RGD loop in the penton base [47,61].

Enteric human adenoviruses HAdV-F40 and HAdV-F41 have two fiber genes of different length, but only incorporate one fiber per vertex, either a long or a short one [64]. All known Aviadenoviruses have two fibers per vertex [65]. Exceptionally, in the lizard Atadenovirus LAdV-2 two fiber genes were found, assembled as either one short or three long fiber projections per vertex [66]. It is to be hoped that the new cryo-EM methods will also provide information on these more complex architectures in a not so distant future.

### 3.4. Protein IIIa

Witness to the complexity of the adenovirus capsid, and the challenges it has posed for structural biology, are the numerous changes in the position assigned to protein IIIa (reviewed in [9]). This protein went from spanning the capsid shell at the edges near the 2-fold icosahedral symmetry [67], to occupying the inner vertex region [68]. When near atomic resolution was achieved, a crystallographic study returned IIIa to an external position at the edges [69], while the cryo-EM analysis kept it located underneath the penton [7]. As more structural data have become available, the internal position of protein IIIa has been confirmed and is no longer a source of debate [8,28,29].

There are five copies of IIIa underneath each vertex (Figure 1b). Protein IIIa has 585 amino acids in HAdV-C5, but only residues 7 to 300 have been traced in the high-resolution map [28]. The rest of the polypeptide chain is not icosahedrally ordered and remains undefined. The traced part of IIIa has a predominantly α-helical fold and consists of two globular domains connected by a long α-helix. The N-terminal domain was termed the GOS-glue domain, as it connects the penton and peripentonal hexons, keeping the structure of the GOS together (Figure 1b) [7]. In the human adenovirus structures, the C-terminal domain binds protein VIII, which helps joining the GOS to the GON hexons.

In spite of being among the core genes conserved throughout the Adenoviridae family [35], recently solved structures indicate that there is a conformational variability in this protein associated to the different species and genera. The variability is subtle among the human adenoviruses: In HAdV-D26, an extra domain of protein IIIa was found to be ordered, formed by amino acids 314 to 390 [29]. In HAdV-F41, the helix connecting the GOS-glue and VIII-interacting domains is slightly kinked, and it has been proposed that this kink facilitates additional contacts between IIIa and the N-terminal arm of penton base, stabilizing the penton of the enteric virus [30]. A much more drastic conformational change has been found in the first high-resolution structure of an adenovirus not belonging to the Mastadenovirus genera. In the Atadenovirus LAdV-2, the C-terminal domain of IIIa is rotated around the axis of the connecting helix by more than 200 degrees with respect to its counterpart in HAdV-C5 (Figure 2d). Although the domain fold is very similar to the human adenovirus proteins, this large movement changes completely its interactions with the surrounding molecules, removing contacts with protein VIII beneath the GOS. This large change in Atadenovirus protein IIIa seems to be induced by the presence of an unidentified genus-specific peptide beneath the vertex [31].

### 3.5. Protein VI

Polypeptide VI (250 residues in HAdV-C5) is cleaved at two positions (after residues 33 and 239 in HAdV-C5) by the adenovirus maturation protease AVP [41]. The C-terminal peptide pVI_C_ acts as a cofactor required for AVP activation, in a remarkable one-dimensional chemistry mechanism: pVI_C_ slides on the dsDNA molecule and binds covalently to AVP, which then uses the viral genome as a track to reach all its substrates in the core and the inner capsid surface [41]. Beyond the N-terminal cleavage, a region containing a predicted amphipathic α-helix (residues 34–54) interacts with lipid bilayers altering their curvature, therefore conferring membrane-lytic activity to protein VI. This activity is required for the virion to escape the endosome during entry (reviewed in [70]).

High-resolution structural data on protein VI are scarce. Weak density inside a hexon cavity opening towards the virus core has been assigned to the cleaved N-terminal peptide (pVI_N_, residues 5–33) in HAdV-C5 [28] and HAdV-D26 [29] (Figure 1b). While in HAdV-D26 the pVI_N_ peptide was traced in such a way that the cleavage site was located at the rim of the hexon cavity, accessible to the protease as it slides on the dsDNA, in HAdV-C5 the chain was traced in the opposite direction, in such a way that the cleavage site is hidden inside the hexon cavity and oriented away from the core. However, the available evidence indicates that the correct direction for pVI_N_ is the one modeled in HAdV-C5 [28]. First, the HAdV-C5 model is based in a map with higher resolution than that of HAdV-D26, facilitating the identification of landmark sidechains; second, in a variant where protein VI is not cleaved at the N-terminal site, pVI_N_ as traced in [28] is connected with extra density attributable to the amphipathic, membrane-lytic peptide [71].

In HAdV-C5, residues 109–143 of protein VI were also modeled, closing the cavity of one of the four hexons in the asymmetric unit (Figure 1b) [28]. The fact that density for all the traced pVI fragments is weak, and found only near a few of the hexons, indicates that the protein is not icosahedrally ordered, and that it does not fill all its possible binding sites in hexon. Partial occupancy is expected, as there are 720 hexon monomers in the capsid and approximately 360 copies of protein VI [72], which are too few for a 1:1 pVI:hexon stoichiometry, but too many to have one copy of VI per hexon trimer. It has recently been found that both the unusual pVI:hexon stoichiometry, and the odd location of the pVI_N_ cleavage site in its recessed position inside the hexon cavity, can be understood by considering an unexpected interplay between protein VI and core protein VII during assembly (see Section 4.4) [71].

### 3.6. Protein VIII

Protein VIII (227 residues in HAdV-C5) is cleaved by the maturation protease at three sites [41]. The two larger fragments (residues 2–112 and 157–227) stay together inside the capsid, stabilizing hexon unions on the inner surface of the icosahedral shell. There are two independent copies of protein VIII per asymmetric unit. One of them is wedged between protein IIIa and the peripentonal hexons, collaborating in the GOS–GON union. The second copy is located beneath the GON (Figure 1b). Each copy of protein VIII interacts with four hexon trimers. Some of these interactions consist in a so-called β-sheet augmentation, in which a β-strand in protein VIII is incorporated into one of the jelly rolls in the neighboring hexon trimer [7,31].

The two larger products of protein VIII maturation have been modeled in all available high-resolution structures of adenovirus virions [7,27,28,29,30,31]. The excised central peptides have lower sequence conservation, vary in size among different adenoviruses, and do not seem to follow the icosahedral symmetry. They may play a role in stabilization of the Atadenovirus and enteric human adenovirus capsids, but this proposal was based on poorly defined densities in which no sequence could be unequivocally assigned [30,31]. Additional evidence is required to assess this point.

### 3.7. Protein IX

Protein IX (140 residues in HAdV-C5) is the only minor coat protein located on the outer capsid surface. This protein adopts an elongated conformation composed of three domains: N-terminal, linker (or rope), and C-terminal. The N-terminal domains of three IX molecules associate in a triskelion-shaped feature located in the valleys between hexons at the icosahedral 3-fold axis (I3) and at the local 3-fold symmetry axis formed by hexons 2, 3, and 4 in the asymmetric unit (L3) (Figure 1a,b and Figure 2e). The rope domain is highly flexible, and crawls around the hexons on the capsid surface, forming a sort of hairnet, until it reaches the facet edge [73]. At the facet edges, the C-terminal domains of three IX molecules join a fourth one coming from the neighboring facet, to form a coiled coil with three parallel and one anti-parallel α-helices, in the case of HAdV-C5 and HAdV-D26 [7,28,29]. There are four triskelions, but only three helix bundles, per icosahedron facet (Figure 2e). Protein IX is dispensable for AdV assembly, but IX-deletion mutants assemble viral particles with low thermostability. Importantly, the triskelion domain is enough to confer capsid thermostability [74].

In non-human Mastadenoviruses canine (CAdV-1) [75], bovine (BAdV-3) [32], and bat (BtAdV-250A) [76], the rope domain is shorter, and the C-terminal domains of IX form coiled coils with only three parallel α-helices located directly on top of their N-terminal triskelion at both the I3 and L3 axis, protruding in a radial orientation between the towers of the hexons [77]. In these viruses, there are four triskelions and four helix bundles per icosahedron facet.

Surprisingly, in the enteric HAdV-F41 capsid there is no density corresponding to the C-terminal 4-helix bundle at the icosahedron edges as in HAdV-C5 and HAdV-D26 [27,30]. Blurry density on top of the triskelions at the L3 axis suggests a conformation similar to the non-human Mastadenoviruses, but there is not any density, even weak, which could account for a helix bundle on top of the I3 triskelion. Therefore, the arrangement of the C-terminal domain of HAdV-F41 protein IX does not seem to follow any of the architectures previously observed in human or non-human adenoviruses. A model has been proposed where protein IX in HAdV-F41 would form four triskelions and three mobile helix bundles, located on top of the L3 triskelions, per facet (Figure 2e) [30].

Protein IX is unique to Mastadenoviruses, but the Atadenovirus specific protein LH3 plays a similar architectural role in the capsid [78]. LH3 forms prominent knobs protruding over the towers of hexons at the I3 and L3 icosahedral axes. Notably, these knobs have a trimeric β-helix fold typical of receptor binding bacteriophage tailspikes [26] (Figure 2e). The LH3 trimer is highly stable and has extensive contacts with the surrounding hexons, likely contributing to the high stability of the Atadenovirus capsids [26,31]. The C-termini of proteins LH3, IX in non-human Mastadenoviruses, and presumably in HAdV-F41, are in a location above the hexon towers more exposed than in HAdV-C5, suggesting that they may have advantages as a locale for exogenous peptide fusion in vector design [30,31,77].

Beneath the knobs, LH3 contacts the capsid surface via a triskelion arrangement which is structurally identical to that of protein IX, indicating a capsid-binding element conserved between Atadenoviruses and Mastadenoviruses [31]. This means that on the one hand, LH3 has negligible sequence similarity with IX but a structurally similar N-terminal domain; on the other, LH3 has high sequence similarity to the C-terminal domain of human adenovirus E1B 55K, a non-structural protein whose gene is directly upstream of the gene coding for IX [79]. The combination of structural and sequence analyses indicates that, in the course of adenovirus evolution, a common ancestor of Atadenoviruses and Mastadenoviruses acquired an LH3-like gene from a bacterium or bacteriophage, perhaps by sharing the same environment (e.g., the host gut). In Mastadenoviruses, this gene was duplicated, and each copy evolved independently. One copy replaced the triskelion with a large N-terminal extension, losing the capacity to bind to the capsid and acquiring new functions in the infectious cycle (E1B-55K). The other copy conserved the triskelion and a structural role, but changed its C-terminus drastically, giving rise to protein IX [31].

## 4. Core Proteins

**Figure 3:**
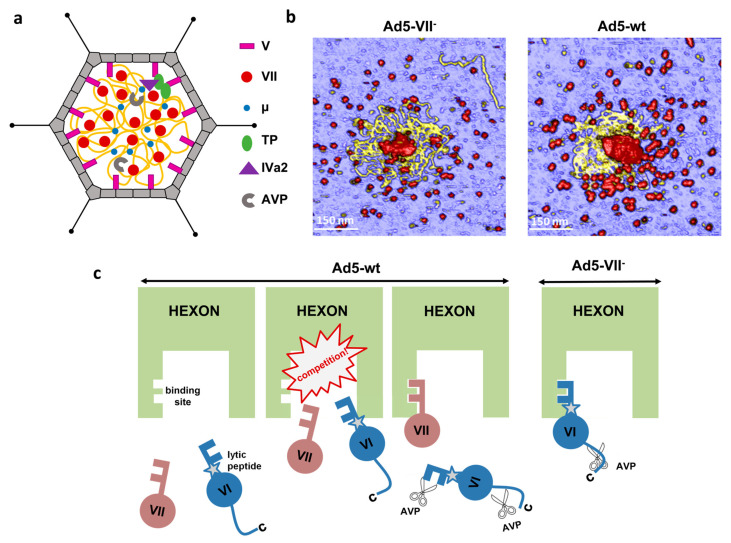
Roles of core protein VII in AdV assembly. (**a**) Schematic representation of the core with the dsDNA in yellow and proteins depicted according to the key at the right. (**b**) AFM images showing the core components released after breakage of Ad5-VII- and Ad5-wt particles. Material with a height consistent with dsDNA is yellow. Protein debris is in red. Reproduced from [91]. (**c**) Competition between proteins VI and VII for hexon binding impinges on AdV maturation and entry. Reproduced from [71].

A distinctive feature of adenoviruses is the incorporation of a large amount (more than 20 MDa) of virus encoded, DNA-binding proteins packed inside the virion together with the genome (Figure 3a). Core proteins V, VII, and µ are highly basic proteins, expected to act as DNA condensing agents [38,39]. There is no structural information for any of them in solution, and the core organization is not revealed by cryo-EM studies using icosahedral symmetry and single particle averaging. Small fragments of proteins VII and V have been modeled in recent cryo-EM studies of HAdV-C5 and HAdV-F41 virions, interacting with the inner surfaces of hexons [27,28].

### 4.1. Protein V

Protein V has 368 amino acids and a predicted isoelectric point (pI) of 10 in HAdV-C5, and is present in approximately 150 copies per virion [72,80]. Cross-linking studies identified the interactions among the core proteins, indicating the formation of dimers of proteins V and VII. Moreover, the same study suggested that protein V exists in a complex with VII and µ, but never found VII and µ interacting without V, indicating that VII and µ are in close contact with V [81]. More recent studies have shown that recombinant protein V exists in a dimer–monomer equilibrium, and there is a direct association between proteins V and VI in solution, supporting a model where protein V bridges the core with the capsid [82]. The majority of protein V is released at the beginning of uncoating [83]. A recent cryo-EM study of HAdV-F41 assigned density located in a pocket formed by hexons 2, 3, and 4 to a central region of protein V (residues 170–194) [27].

Protein V is only present in Mastadenoviruses, and is not essential for adenovirus assembly [84]. Two genus-specific, positively charged proteins, P32k and LH2, have been found in Atadenovirus virions, and proposed to be located on the inner capsid surface, substituting for protein V. However, their position in the viral particle has not been unequivocally identified [26,31,78].

### 4.2. Proteins VII and μ

Protein VII is the most abundant protein in the adenovirus core. Its estimated copy number in the virion varies between 500 and 800 [72,80]. It is rich in arginine amino acids, which confer it a high positive charge (predicted pI 12.35 in HAdV-C5). AVP cleaves the precursor pVII at residues 13 and 24 (from a total of 198 in HAdV-C5) [41]. In the most recent structural analysis of HAdV-C5, it was found that the second cleaved N-terminal peptide (pVII_N2_, residues 14–24) occupies a position equivalent to that of the protein VI N-terminal peptide pVI_N_, lining the hexon cavity wall (Figure 1b) [28]. This observation was unexpected, because as a core protein, no part of VII was thought to be icosahedrally ordered, and there were no previous indications that pVII interacted with hexon. Residues Ser31 in pVI_N_ and Phe22 of pVII_N2_ interact with the same binding pocket in the hexon wall, raising the possibility that pVI and pVII may compete for hexon binding during assembly.

Protein µ (also called protein X) is the smallest core protein, with 80 amino acids in HAdV-C5 and a predicted pI of 12.88. Estimations on its copy number vary between 100 and 300 [72,85]. In HAdV-C5, AVP cleaves the precursor form of µ at residues 32 and 51 [41]. Both VII and μ condense dsDNA in solution [86,87].

### 4.3. Organization of the Adenovirus Core

Early studies showed that cores released from HAdV-C5 particles under mild disruption conditions contain proteins VII, V, and µ, and may form 150–300 Å-thick fibers. After high ionic strength treatment of these cores, only polypeptide VII remains associated with the viral DNA, forming a “bead-on-a-string” filament reminiscent of cell chromatin, as observed by electron microscopy of metal shadowed specimens [88,89]. A ~120 Å-thick fiber has also been observed in disrupted immature adenovirus particles, which contain the precursor version of proteins VII and μ [90]. Based on these observations, it has been proposed that protein VII may condense dsDNA by wrapping it around the protein, while μ may exert a bridging action between different regions of the dsDNA molecule [39]. Discerning the organization of the adenovirus core in its physiological context, i.e., inside the capsid, is not straightforward. Cryo-EM high-resolution studies rely on averaging thousands of copies of the same macromolecular object, and it is not clear yet if the core architecture is the same in each viral particle. Cryo-electron tomography yields three-dimensional maps of unique objects, but averaging is still required to achieve a certain level of detail.

A first glimpse on the adenovirus core organization has been provided by a cryo-electron tomography study combined with molecular dynamics simulations. This study indicated that the HAdV-C5 genome and core proteins can be modeled as an ensemble of soft particles related to each other by a soft electrostatic repulsion. This soft repulsion would be generated by an excess of DNA negative charges not screened by the positive charges of the core proteins, and generates a certain degree of internal pressure in the adenovirus particle [39]. Atomic force microscopy (AFM) studies comparing the mechanical properties of HAdV-C5 virions and particles lacking core protein VII have recently confirmed that the major core protein plays a role in decreasing the electrostatic repulsion created by confinement of the dsDNA genome, therefore modulating the internal pressure in the capsid [91]. The AFM images also showed the aspect of core contents released upon mechanical breakage of the particles in physiological conditions, i.e., in liquid buffer (as opposed to previous studies on dehydrated, Pt-C shadowed specimens) (Figure 3b). In these conditions, it was observed that different regions of the dsDNA molecule associate in bundles interspersed with clusters presumably formed by DNA wrapping around protein VII. It was concluded that protein VII condenses the adenovirus genome by combining direct clustering and promotion of bridging by other core proteins, i.e., protein μ [91].

### 4.4. Role of the Core Proteins in Adenovirus Assembly, Maturation and Entry

Core proteins have long been considered condensing agents required to tightly pack the adenovirus genome within the capsid. However, the volume relation between the interior of the adenovirus particle and the genome is much less tight than in other dsDNA viruses, for example herpesvirus [38], and HAdV-C5 particles lacking protein VII (Ad5-VII-) can be assembled, showing that the protein condensing action is not required for genome packaging [92]. Structural and biophysical studies are revealing additional roles for core proteins in the adenovirus infectious cycle.

Immature adenovirus virions produced by the HAdV-C2 *ts1* mutant, which contain the precursor versions of all AVP targets including core proteins VII and μ, do not release protein VI in the early endosome, become trapped in the endocytic pathway, and are eventually destroyed in lysosomes [70]. Comparison of the structure, stability, and mechanical properties of mature and immature particles indicates that maturation of the core proteins causes an increase in the internal pressure of adenovirus virions, presumably due to a change in their interactions with the dsDNA molecule upon proteolytic cleavage. The higher pressure turns the mature particle metastable, facilitating penton loss in the initial stages of uncoating, which opens the way for protein VI to be exposed and carry out its membrane lytic function in the endosome [90,93,94,95]. Thus, maturation of core proteins plays a crucial role to ensure correct uncoating, and therefore infectivity.

When protein VII is absent, non-infectious particles are assembled. Ad5-VII particles do not expose protein VI and become trapped in the endosome [71,92]. Unlike immature *ts1* particles, Ad5-VII-particles have higher internal pressure than the mature virion [91] and have no trouble releasing pentons, as shown by thermal and mechanical stability assays [71]. Instead, these particles fail to expose the lytic protein because its N-terminal region, which includes the pVI_N_ peptide and the amphipathic α-helix, remains hidden inside the hexon cavity oriented towards the virus core, unavailable for proteolytic cleavage and interaction with the endosome membrane [28,71,92]. These observations support a model where the N-terminal regions of proteins VI (360 copies) and VII (500–800 copies) compete for hexon binding sites (720, one per hexon monomer) during adenovirus assembly. The precursor proteins pVI or pVII would continually be pushed out from the hexon cavity by their competitors, putting them in the path of AVP sliding on the DNA. In the absence of protein VII, the competition does not exist, and all pVI copies remain secured inside the hexon cavity, with their pVI_N_ cleavage site hidden away from the protease and the lytic peptide shielded by hexon, preventing virion escape from the endosome (Figure 3c) [71].

## 5. Conclusions

Advances in structural biology methods (notably cryo-EM), availability of novel techniques (such as AFM), and discovery of new viruses have resulted in notable advances in our understanding of the adenovirus particle organization and its variations throughout the different species and genera. However, many questions remain open. We now know that core proteins are not just passive players helping squeeze a stiff dsDNA molecule inside a small space, but they play active roles in assembly, maturation and uncoating, defining the capacity of adenovirus virions to deliver their genome in the cell [71,90,91,93,94,95,96]. Yet, there are no structural data on any of the core proteins, and only initial glimpses on the core architecture have been obtained by cryo-electron tomography and atomic force microscopy imaging [39,91]. We still do not understand how the genome, heavily bound by core proteins, associates with the capsid shell, and there are only limited data on the organization of the packaging machinery [42,97,98]. We have many more structures of fiber heads, alone or receptor bound (Table 1), and data indicating new receptor binding strategies [12,20,53,99]. Virion structures show that the external decorating proteins IX and LH3 mark adenovirus evolution; these proteins may play a role in host specificity, and constitute interesting locations for exogenous peptide display or retargeting [7,27,28,29,30,31,77]. Yet, more information is required to understand the relation between adenovirus types and their related pathologies. In spite of their being old friends (or foes) [100], adenoviruses keep a large amount of secrets that scientists will need to unveil. This is necessary if we want to efficiently fight adenovirus caused infections, or tailor the virus to suit our needs in the battle against other diseases.

## Figures and Tables

**Table 1:** Selected adenovirus-related high-resolution structures solved since 2010.

Specimen	Virus Type ^1^	Genus	PDB ^2^ ID	Reference
Fiber knob	HAdV-C5	Mastadenovirus	6HCN	[10]
Fiber knob	HAdV-D10	Mastadenovirus	6QPM	unpublished
Fiber knob	HAdV-D30	Mastadenovirus	6STU	[11]
Fiber knob	HAdV-D48	Mastadenovirus	6FJQ	[10]
Fiber knob	HAdV-D49	Mastadenovirus	6QPN	[11]
Fiber knob	HAdV-D56	Mastadenovirus	7AJP	[12]
Fiber knob	RAdV-1	Siadenovirus	5FJL	[13]
Fiber knob	TAdV-3	Siadenovirus	4CW8	[14]
Fiber knob	BAdV-4	Atadenovirus	4UE0	[15]
Fiber knob	DAdV-1	Atadenovirus	6ITX	[16]
Fiber knob	SnAdV-1	Atadenovirus	4D0V	[17]
Fiber knob with CD46	HAdV-B21	Mastadenovirus	3L89	[18]
Fiber knob with sialic acid	HAdV-D26	Mastadenovirus	6QU8	[19]
Fiber knob with desmoglein 2	HAdV-B3	Mastadenovirus	6QNT	[20]
Fiber knob with desmoglein 2	HAdV-B7	Mastadenovirus	7AGF	[21]
Fiber knob with trivalent sialic acid inhibitor	HAdV-D37	Mastadenovirus	4XQA	[22]
Short fiber knob with 2-O-Methyl-5-N-Acetylneuraminic acid	HAdV-G52	Mastadenovirus	4XL8	[23]
Short fiber knob with α-(2,8)-trisialic acid	HAdV-G52	Mastadenovirus	6G47	[24]
Fiber knob and shaft with N-acetylglucosamine	MAdV-2	Mastadenovirus	5NC1	[25]
Protein LH3	SnAdV-1	Atadenovirus	5G5O	[26]
Penton base	HAdV-F41	Mastadenovirus	6Z7Q	[27]
Viral particle	HAdV-C5	Mastadenovirus	6B1T	[28]
Viral particle	HAdV-C5	Mastadenovirus	6CGV	[8]
Viral particle	HAdV-D26	Mastadenovirus	5TX1	[29]
Viral particle	HAdV-F41	Mastadenovirus	6YBA	[30]
Viral particle	HAdV-F41	Mastadenovirus	6Z7N	[27]
Viral particle	LAdV-2	Atadenovirus	6QI5	[31]
Viral particle	BAdV-3	Mastadenovirus	3ZIF	[32]

^1^ HAdV: human adenovirus. RAdV: raptor adenovirus. TAdV: turkey adenovirus. BAdV: bovine adenovirus. DAdV: duck adenovirus. MAdV: mouse adenovirus. SnAdV: snake adenovirus. LAdV: lizard adenovirus. ^2^ Protein Data Bank, http://www.pdb.org/ (accessed on 25 January 2021).

## References

[1] Rowe W.P., Huebner R.J., Gilmore L.K., Parrott R.H., Ward T.G. (1953). Isolation of a cytopathogenic agent from human adenoids undergoing spontaneous degeneration in tissue culture. Proc. Soc. Exp. Biol. Med..

[2] Harrach B., Tarjan Z.L., Benko M. (2019). Adenoviruses across the animal kingdom: A walk in the zoo. FEBS Lett..

[3] Berk A.J., Knipe D.M., Howley P.M. (2013). Adenoviridae. Fields Virology.

[4] Kremer E.J. (2020). Pros and cons of adenovirus-based SARS-CoV-2 vaccines. Mol. Ther. J. Am. Soc. Gene Ther..

[5] Tumban E. (2020). Lead SARS-CoV-2 candidate vaccines: Expectations from phase III trials and recommendations post-vaccine approval. Viruses.

[6] Hasanpourghadi M., Novikov M., Ertl H.C.J. (2021). COVID-19 Vaccines Based on Adenovirus Vectors. Trends Biochem Sci.

[7] Liu H., Jin L., Koh S.B., Atanasov I., Schein S., Wu L., Zhou Z.H. (2010). Atomic structure of human adenovirus by cryo-EM reveals interactions among protein networks. Science.

[8] Kundhavai Natchiar S., Venkataraman S., Mullen T.M., Nemerow G.R., Reddy V.S. (2018). Revised crystal structure of human adenovirus reveals the limits on protein IX quasi-equivalence and on analyzing large macromolecular complexes. J. Mol. Biol..

[9] San Martín C. (2012). Latest insights on adenovirus structure and assembly. Viruses.

[10] Baker A.T., Greenshields-Watson A., Coughlan L., Davies J.A., Uusi-Kerttula H., Cole D.K., Rizkallah P.J., Parker A.L. (2019). Diversity within the adenovirus fiber knob hypervariable loops influences primary receptor interactions. Nat. Commun..

[11] Baker A.T., Davies J.A., Bates E.A., Moses E., Mundy R.M., Marlow G., Cole D.K., Bliss C.M., Rizkallah P.J., Parker A.L. (2021). The fiber knob protein of human adenovirus type 49 mediates highly efficient and promiscuous infection of cancer cell lines using a novel cell entry mechanism. J. Virol..

[12] Persson B.D., John L., Rafie K., Strebl M., Frangsmyr L., Ballmann M.Z., Mindler K., Havenga M., Lemckert A., Stehle T. (2021). Human species D adenovirus hexon capsid protein mediates cell entry through a direct interaction with CD46. Proc. Natl. Acad. Sci. USA.

[13] Nguyen T.H., Ballmann M.Z., Do H.T., Truong H.N., Benkő M., Harrach B., van Raaij M.J. (2016). Crystal structure of raptor adenovirus 1 fibre head and role of the beta-hairpin in siadenovirus fibre head domains. Virol. J..

[14] Singh A.K., Berbís M.Á., Ballmann M.Z., Kilcoyne M., Menéndez M., Nguyen T.H., Joshi L., Cañada F.J., Jiménez-Barbero J., Benkő M. (2015). Structure and sialyllactose binding of the carboxy-terminal head domain of the fibre from a siadenovirus, Turkey adenovirus 3. PLoS ONE.

[15] Nguyen T.H., Vidovszky M.Z., Ballmann M.Z., Sanz-Gaitero M., Singh A.K., Harrach B., Benko M., Van Raaij M.J. (2015). Crystal structure of the fibre head domain of bovine adenovirus 4, a ruminant atadenovirus. Virol. J..

[16] Song Y., Wei Q., Liu Y., Feng H., Chen Y., Wang Y., Bai Y., Xing G., Deng R., Zhang G. (2019). Unravelling the receptor binding property of egg drop syndrome virus (EDSV) from the crystal structure of EDSV fiber head. Int. J. Biol. Macromol..

[17] Singh A.K., Menéndez-Conejero R., San Martín C., van Raaij M.J. (2014). Crystal structure of the fibre head domain of the atadenovirus snake adenovirus 1. PLoS ONE.

[18] Cupelli K., Müller S., Persson B.D., Jost M., Arnberg N., Stehle T. (2010). Structure of adenovirus type 21 knob in complex with CD46 reveals key differences in receptor contacts among species B adenoviruses. J. Virol..

[19] Baker A.T., Mundy R.M., Davies J.A., Rizkallah P.J., Parker A.L. (2019). Human adenovirus type 26 uses sialic acid-bearing glycans as a primary cell entry receptor. Sci. Adv..

[20] Vassal-Stermann E., Effantin G., Zubieta C., Burmeister W., Iseni F., Wang H., Lieber A., Schoehn G., Fender P. (2019). CryoEM structure of adenovirus type 3 fibre with desmoglein 2 shows an unusual mode of receptor engagement. Nat. Commun..

[21] Hograindleur M.-A., Effantin G., Fenel D., Mas C., Lieber A., Schoehn G., Fender P., Vassal-Stermann E. (2020). Binding mechanism elucidation of the acute respiratory disease causing agent adenovirus of serotype 7 to desmoglein-2. Viruses.

[22] Caraballo R., Saleeb M., Bauer J., Liaci A.M., Chandra N., Storm R.J., Frängsmyr L., Qian W., Stehle T., Arnberg N. (2015). Triazole linker-based trivalent sialic acid inhibitors of adenovirus type 37 infection of human corneal epithelial cells. Org. Biomol. Chem..

[23] Lenman A., Liaci A.M., Liu Y., Ardahl C., Rajan A., Nilsson E., Bradford W., Kaeshammer L., Jones M.S., Frangsmyr L. (2015). Human adenovirus 52 uses sialic acid-containing glycoproteins and the coxsackie and adenovirus receptor for binding to target cells. PLoS Pathog..

[24] Lenman A., Liaci A.M., Liu Y., Frängsmyr L., Frank M., Blaum B.S., Chai W., Podgorski I.I., Harrach B., Benkő M. (2018). Polysialic acid is a cellular receptor for human adenovirus 52. Proc. Natl. Acad. Sci. USA.

[25] Singh A.K., Nguyen T.H., Vidovszky M.Z., Harrach B., Benkő M., Kirwan A., Joshi L., Kilcoyne M., Berbis M.Á., Cañada F.J. (2018). Structure and N-acetylglucosamine binding of the distal domain of mouse adenovirus 2 fibre. J. Gen. Virol..

[26] Menéndez-Conejero R., Nguyen T.H., Singh A.K., Condezo G.N., Marschang R.E., van Raaij M.J., San Martín C. (2017). Structure of a reptilian adenovirus reveals a phage tailspike fold stabilizing a vertebrate virus capsid. Structure.

[27] Rafie K., Lenman A., Fuchs J., Rajan A., Arnberg N., Carlson L.-A. (2021). The structure of enteric human adenovirus 41—A leading cause of diarrhea in children. Sci. Adv..

[28] Dai X., Wu L., Sun R., Zhou Z.H. (2017). Atomic structures of minor proteins VI and VII in human adenovirus. J. Virol..

[29] Yu X., Veesler D., Campbell M.G., Barry M.E., Asturias F.J., Barry M.A., Reddy V.S. (2017). Cryo-EM structure of human adenovirus D26 reveals the conservation of structural organization among human adenoviruses. Sci. Adv..

[30] Pérez-Illana M., Martinez M., Condezo G.N., Hernando-Pérez M., Mangroo C., Brown M., Marabini R., San Martín C. (2021). Cryo-EM structure of enteric adenovirus HAdV-F41 highlights structural variations among human adenoviruses. Sci. Adv..

[31] Marabini R., Condezo G.N., Krupovic M., Menéndez-Conejero R., Gómez-Blanco J., San Martín C. (2021). Near atomic structure of an atadenovirus reveals a conserved capsid-binding motif and intergenera variations in cementing proteins. Sci. Adv..

[32] Cheng L., Huang X., Li X., Xiong W., Sun W., Yang C., Zhang K., Wang Y., Liu H., Huang X. (2014). Cryo-EM structures of two bovine adenovirus type 3 intermediates. Virology.

[33] Davison A.J., Wright K.M., Harrach B. (2000). DNA sequence of frog adenovirus. J. Gen. Virol..

[34] Doszpoly A., Harrach B., LaPatra S., Benko M. (2019). Unconventional gene arrangement and content revealed by full genome analysis of the white sturgeon adenovirus, the single member of the genus Ichtadenovirus. Infect. Genet. Evol..

[35] Davison A.J., Benkő M., Harrach B. (2003). Genetic content and evolution of adenoviruses. J. Gen. Virol..

[36] Goddard T.D., Huang C.C., Meng E.C., Pettersen E.F., Couch G.S., Morris J.H., Ferrin T.E. (2018). UCSF ChimeraX: Meeting modern challenges in visualization and analysis. Protein Sci..

[37] Pettersen E.F., Goddard T.D., Huang C.C., Couch G.S., Greenblatt D.M., Meng E.C., Ferrin T.E. (2004). UCSF Chimera—A visualization system for exploratory research and analysis. J. Comput. Chem..

[38] Marion S., San Martín C., Siber A. (2017). Role of condensing particles in polymer confinement: A model for virus-packed “minichromosomes”. Biophys. J..

[39] Pérez-Berná A.J., Marion S., Chichón F.J., Fernández J.J., Winkler D.C., Carrascosa J.L., Steven A.C., Šiber A., San Martín C. (2015). Distribution of DNA-condensing protein complexes in the adenovirus core. Nucleic Acids Res..

[40] Hoeben R.C., Uil T.G. (2013). Adenovirus DNA replication. Cold Spring Harb. Perspect. Biol..

[41] Mangel W.F., San Martín C. (2014). Structure, function and dynamics in adenovirus maturation. Viruses.

[42] Ahi Y.S., Mittal S.K. (2016). Components of adenovirus genome packaging. Front. Microbiol..

[43] Burnett R.M., Grütter M.G., White J.L. (1985). The structure of the adenovirus capsid. I. An envelope model of hexon at 6 A resolution. J. Mol. Biol..

[44] Condezo G.N., Martín-González N., Pérez-Illana M., Hernando-Pérez M., Gallardo J., San Martín C. (2021). Adenoviruses (Adenoviridae) and their structural relatives. Encyclopedia of Virology.

[45] Crawford-Miksza L., Schnurr D.P. (1996). Analysis of 15 adenovirus hexon proteins reveals the location and structure of seven hypervariable regions containing serotype-specific residues. J. Virol..

[46] Caspar D.L.D., Klug A. (1962). Physical principles in the construction of regular viruses. Cold Spring Harb Symp Quant Biol.

[47] Abrishami V., Ilca S.L., Gomez-Blanco J., Rissanen I., de la Rosa-Trevin J.M., Reddy V.S., Carazo J.M., Huiskonen J.T. (2021). Localized reconstruction in Scipion expedites the analysis of symmetry mismatches in cryo-EM data. Prog. Biophys. Mol. Biol..

[48] Ebner K., Pinsker W., Lion T. (2005). Comparative sequence analysis of the hexon gene in the entire spectrum of human adenovirus serotypes: Phylogenetic, taxonomic, and clinical implications. J. Virol..

[49] Diaz K., Hu C.T., Sul Y., Bromme B.A., Myers N.D., Skorohodova K.V., Gounder A.P., Smith J.G. (2020). Defensin-driven viral evolution. PLoS Pathog..

[50] Alba R., Bradshaw A.C., Parker A.L., Bhella D., Waddington S.N., Nicklin S.A., van Rooijen N., Custers J., Goudsmit J., Barouch D.H. (2009). Identification of coagulation factor (F)X binding sites on the adenovirus serotype 5 hexon: Effect of mutagenesis on FX interactions and gene transfer. Blood.

[51] Zubieta C., Schoehn G., Chroboczek J., Cusack S. (2005). The structure of the human adenovirus 2 penton. Mol. Cell.

[52] Albinsson B., Kidd A.H. (1999). Adenovirus type 41 lacks an RGD alpha(v)-integrin binding motif on the penton base and undergoes delayed uptake in A549 cells. Virus Res..

[53] Rajan A., Persson B.D., Frangsmyr L., Olofsson A., Sandblad L., Heino J., Takada Y., Mould A.P., Schnapp L.M., Gall J. (2018). Enteric species F human adenoviruses use laminin-binding integrins as co-receptors for infection of Ht-29 cells. Sci. Rep..

[54] Madisch I., Hofmayer S., Moritz C., Grintzalis A., Hainmueller J., Pring-Akerblom P., Heim A. (2007). Phylogenetic analysis and structural predictions of human adenovirus penton proteins as a basis for tissue-specific adenovirus vector design. J. Virol..

[55] Besson S., Vragniau C., Vassal-Stermann E., Dagher M.C., Fender P. (2020). The adenovirus dodecahedron: Beyond the platonic story. Viruses.

[56] Stasiak A.C., Stehle T. (2020). Human adenovirus binding to host cell receptors: A structural view. Med. Microbiol. Immunol..

[57] Nicklin S.A., Wu E., Nemerow G.R., Baker A.H. (2005). The influence of adenovirus fiber structure and function on vector development for gene therapy. Mol. Ther. J. Am. Soc. Gene Ther..

[58] Van Raaij M.J., Mitraki A., Lavigne G., Cusack S. (1999). A triple beta-spiral in the adenovirus fibre shaft reveals a new structural motif for a fibrous protein. Nature.

[59] Wu E., Pache L., Von Seggern D.J., Mullen T.M., Mikyas Y., Stewart P.L., Nemerow G.R. (2003). Flexibility of the adenovirus fiber is required for efficient receptor interaction. J Virol.

[60] Li J., Lad S., Yang G., Luo Y., Iacobelli-Martinez M., Primus F.J., Reisfeld R.A., Li E. (2006). Adenovirus Fiber Shaft Contains a Trimerization Element That Supports Peptide Fusion for Targeted Gene Delivery. Journal of Virology.

[61] Liu H., Wu L., Zhou Z.H. (2011). Model of the trimeric fiber and its interactions with the pentameric penton base of human adenovirus by cryo-electron microscopy. J. Mol. Biol..

[62] Cao C., Dong X., Wu X., Wen B., Ji G., Cheng L., Liu H. (2012). Conserved fiber-penton base interaction revealed by nearly atomic resolution cryo-electron microscopy of the structure of adenovirus provides insight into receptor interaction. J. Virol..

[63] Stass R., Ilca S.L., Huiskonen J.T. (2018). Beyond structures of highly symmetric purified viral capsids by cryo-EM. Curr. Opin. Struct. Biol..

[64] Kidd A.H., Chroboczek J., Cusack S., Ruigrok R.W. (1993). Adenovirus type 40 virions contain two distinct fibers. Virology.

[65] Hess M., Cuzange A., Ruigrok R.W.H., Chroboczek J., Jacrot B. (1995). The avian adenovirus penton: Two fibres and one base. J. Mol. Biol..

[66] Pénzes J.J., Menéndez-Conejero R., Condezo G.N., Ball I., Papp T., Doszpoly A., Paradela A., Pérez-Berná A.J., López-Sanz M., Nguyen T.H. (2014). Molecular characterization of a lizard adenovirus reveals the first atadenovirus with two fiber genes and the first adenovirus with either one short or three long fibers per penton. J. Virol..

[67] Stewart P.L., Fuller S.D., Burnett R.M. (1993). Difference imaging of adenovirus: Bridging the resolution gap between X-ray crystallography and electron microscopy. EMBO J..

[68] Saban S.D., Silvestry M., Nemerow G.R., Stewart P.L. (2006). Visualization of alpha-helices in a 6-angstrom resolution cryoelectron microscopy structure of adenovirus allows refinement of capsid protein assignments. J. Virol..

[69] Reddy V.S., Nemerow G.R. (2014). Structures and organization of adenovirus cement proteins provide insights into the role of capsid maturation in virus entry and infection. Proc. Natl. Acad. Sci. USA.

[70] Greber U.F., Flatt J.W. (2019). Adenovirus entry: From infection to immunity. Annu. Rev. Virol..

[71] Hernando-Pérez M., Martín-González N., Pérez-Illana M., Suomalainen M., Condezo G.N., Ostapchuk P., Gallardo J., Menéndez M., Greber U.F., Hearing P. (2020). Dynamic competition for hexon binding between core protein VII and lytic protein VI promotes adenovirus maturation and entry. Proc. Natl. Acad. Sci. USA.

[72] Benevento M., Di Palma S., Snijder J., Moyer C.L., Reddy V.S., Nemerow G.R., Heck A.J. (2014). Adenovirus composition, proteolysis, and disassembly studied by in-depth qualitative and quantitative proteomics. J. Biol. Chem..

[73] Reddy V.S. (2017). The role of hexon protein as a molecular mold in patterning the protein IX organization in human adenoviruses. J. Mol. Biol..

[74] Vellinga J., van den Wollenberg D.J., van der Heijdt S., Rabelink M.J., Hoeben R.C. (2005). The coiled-coil domain of the adenovirus type 5 protein IX is dispensable for capsid incorporation and thermostability. J. Virol..

[75] Schoehn G., El Bakkouri M., Fabry C.M., Billet O., Estrozi L.F., Le L., Curiel D.T., Kajava A.V., Ruigrok R.W., Kremer E.J. (2008). Three-dimensional structure of canine adenovirus serotype 2 capsid. J. Virol..

[76] Hackenbrack N., Rogers M.B., Ashley R.E., Keel M.K., Kubiski S.V., Bryan J.A., Ghedin E., Holmes E.C., Hafenstein S.L., Allison A.B. (2016). Evolution and cryo-EM capsid structure of a North American bat adenovirus and its relationship to other mastadenoviruses. J. Virol..

[77] Matteson N.L., Barry M.A., Reddy V.S. (2018). Structure-based assessment of protein-protein interactions and accessibility of protein IX in adenoviruses with implications for antigen display. Virology.

[78] Pantelic R.S., Lockett L.J., Rothnagel R., Hankamer B., Both G.W. (2008). Cryoelectron microscopy map of Atadenovirus reveals cross-genus structural differences from human adenovirus. J. Virol..

[79] Gorman J.J., Wallis T.P., Whelan D.A., Shaw J., Both G.W. (2005). LH3, a “homologue” of the mastadenoviral E1B 55-kDa protein is a structural protein of atadenoviruses. Virology.

[80] Van Oostrum J., Burnett R.M. (1985). Molecular composition of the adenovirus type 2 virion. J. Virol..

[81] Chatterjee P.K., Vayda M.E., Flint S.J. (1985). Interactions among the three adenovirus core proteins. J. Virol..

[82] Pérez-Vargas J., Vaughan R.C., Houser C., Hastie K.M., Kao C.C., Nemerow G.R. (2014). Isolation and characterization of the DNA and protein binding activities of adenovirus core protein V. J. Virol..

[83] Puntener D., Engelke M.F., Ruzsics Z., Strunze S., Wilhelm C., Greber U.F. (2011). Stepwise loss of fluorescent core protein V from human adenovirus during entry into cells. J. Virol..

[84] Ugai H., Borovjagin A.V., Le L.P., Wang M., Curiel D.T. (2007). Thermostability/infectivity defect caused by deletion of the core protein V gene in human adenovirus type 5 is rescued by thermo-selectable mutations in the core protein X precursor. J. Mol. Biol..

[85] Hosokawa K., Sung M.T. (1976). Isolation and characterization of an extremely basic protein from adenovirus type 5. J. Virol..

[86] Johnson J.S., Osheim Y.N., Xue Y., Emanuel M.R., Lewis P.W., Bankovich A., Beyer A.L., Engel D.A. (2004). Adenovirus protein VII condenses DNA, represses transcription, and associates with transcriptional activator E1A. J. Virol..

[87] Anderson C.W., Young M.E., Flint S.J. (1989). Characterization of the adenovirus 2 virion protein, mu. Virology.

[88] Vayda M.E., Rogers A.E., Flint S.J. (1983). The structure of nucleoprotein cores released from adenovirions. Nucleic Acids Res..

[89] Mirza M.A., Weber J. (1982). Structure of adenovirus chromatin. Biochim. Biophys. Acta.

[90] Pérez-Berná A.J., Marabini R., Scheres S.H.W., Menéndez-Conejero R., Dmitriev I.P., Curiel D.T., Mangel W.F., Flint S.J., San Martín C. (2009). Structure and uncoating of immature adenovirus. J. Mol. Biol..

[91] Martín-González N., Hernando-Pérez M., Condezo G.N., Pérez-Illana M., Šiber A., Reguera D., Ostapchuk P., Hearing P., San Martín C., de Pablo P.J. (2019). Adenovirus major core protein condenses DNA in clusters and bundles, modulating genome release and capsid internal pressure. Nucleic Acids Res..

[92] Ostapchuk P., Suomalainen M., Zheng Y., Boucke K., Greber U.F., Hearing P. (2017). The adenovirus major core protein VII is dispensable for virion assembly but is essential for lytic infection. PLoS Pathog..

[93] Ortega-Esteban A., Condezo G.N., Pérez-Berná A.J., Chillón M., Flint S.J., Reguera D., San Martín C., de Pablo P.J. (2015). Mechanics of viral chromatin reveals the pressurization of human adenovirus. ACS Nano.

[94] Ortega-Esteban A., Pérez-Berná A.J., Menéndez-Conejero R., Flint S.J., San Martín C., de Pablo P.J. (2013). Monitoring dynamics of human adenovirus disassembly induced by mechanical fatigue. Sci. Rep..

[95] Pérez-Berná A.J., Ortega-Esteban A., Menéndez-Conejero R., Winkler D.C., Menéndez M., Steven A.C., Flint S.J., de Pablo P.J., San Martín C. (2012). The role of capsid maturation on adenovirus priming for sequential uncoating. J. Biol. Chem..

[96] Ortega-Esteban A., Bodensiek K., San Martín C., Suomalainen M., Greber U.F., de Pablo P.J., Schaap I.A. (2015). Fluorescence tracking of genome release during mechanical unpacking of single viruses. ACS Nano.

[97] Condezo G.N., Marabini R., Ayora S., Carazo J.M., Alba R., Chillón M., San Martín C. (2015). Structures of adenovirus incomplete particles clarify capsid architecture and show maturation changes of packaging protein L1 52/55k. J. Virol..

[98] Condezo G.N., San Martín C. (2017). Localization of adenovirus morphogenesis players, together with visualization of assembly intermediates and failed products, favor a model where assembly and packaging occur concurrently at the periphery of the replication center. PLoS Pathog..

[99] Vassal-Stermann E., Mottet M., Ducournau C., Iseni F., Vragniau C., Wang H., Zubieta C., Lieber A., Fender P. (2018). Mapping of Adenovirus of serotype 3 fibre interaction to desmoglein 2 revealed a novel ‘non-classical’ mechanism of viral receptor engagement. Sci. Rep..

[100] Gonçalves M.A.F.V., de Vries A.A.F. (2006). Adenovirus: From foe to friend. Rev. Med. Virol..

